# The Quality, Intake, and Digestibility of Virginia Fanpetals (*Sida* *hermaphrodita* L. Rusby) Silage Produced under Different Technologies and Its Effect on the Performance of Young Cattle

**DOI:** 10.3390/ani11082270

**Published:** 2021-07-31

**Authors:** Cezary Purwin, Maciej Starczewski, Marta Borsuk, Zenon Nogalski, Paulina M. Opyd, Magdalena Mazur-Kuśnirek, Ireneusz Białobrzewski

**Affiliations:** 1 Department of Animal Nutrition and Feed Science, University of Warmia and Mazury in Olsztyn, Oczapowskiego 5, 10-719 Olsztyn, Poland; maciejstarczewski@wp.pl (M.S.); marta.borsuk@uwm.edu.pl (M.B.); paulina.opyd@uwm.edu.pl (P.M.O.); magdalena.mazur@uwm.edu.pl (M.M.-K.); 2 Department of Cattle Breeding and Milk Evaluation, University of Warmia and Mazury in Olsztyn, Oczapowskiego 5, 10-719 Olsztyn, Poland; zenon.nogalski@uwm.edu.pl; 3 Department of Systems Engineering, University of Warmia and Mazury in Olsztyn, Heweliusza 14, 10-718 Olsztyn, Poland; irekb@uwm.edu.pl

**Keywords:** *Sida hermaphrodita* silage, Virginia fanpetals silage, mechanical processing, wilting, intake, digestibility, performance

## Abstract

**Simple Summary:**

Competition for high-quality arable land has prompted a search for alternative forage crops. For example, Virginia fanpetals is a high-yielding perennial plant with low soil requirements. Due to its chemical composition, it can be a valuable component of ruminant diets. Previous research has shown that it cannot be used for hay production, and the natural moisture content of silage was unsatisfactory. Laboratory analyses suggest that the quality and feed value of Virginia fanpetals silage may be affected by harvesting and preservation methods. However, to date, most studies have focused on the nutritional value of Virginia fanpetals silage and its inclusion in animal diets. Therefore, the present study investigated different harvesting and preservation methods, such as direct-cut harvesting with a forage harvester, and harvesting after field wilting with a forage harvester or a round baler. The quality parameters, intake and digestibility of silage, and the performance of growing bulls, fed the experimental silage, as the sole forage was evaluated. It was discovered that the highest-quality silage was produced when herbage was harvested with a forage harvester after field wilting.

**Abstract:**

Different harvesting and preservation methods of Virginia fanpetals herbage were evaluated, based on the chemical composition and digestible organic matter (OM) content (D-value) of silage fed to adult sheep, the intake and digestibility of silage, and the performance of young cattle. The following harvesting methods were compared: direct-cut harvesting with a precision-cut forage harvester (DC), harvesting after field wilting with a precision-cut forage harvester (WC) or a round baler (WRB). The silage was fed for 81 days to 24 Polish Holstein Friesian (HF) bulls, as the sole forage supplemented with 3.0 kg of concentrate/head/day. Harvesting methods affected the density (*p* < 0.001) and water-soluble carbohydrate (WSC) content (*p* = 0.047). Differences were found among the groups in the digestibility coefficients of OM (DC-73.7, WC-78.9, WRB-79.9%) (*p* = 0.007), and crude protein (CP) (69.8%, 77.1%, 78.5%, respectively) (*p* < 0.001). Dry matter intake (DMI) reached 8.38 kg (DC), 8.74 kg (WC) and 7.21 kg (WRB). Live weight gain (LWG) differed (*p* < 0.001) among groups (0.939, 1.033, 0.813 kg/day, respectively). The feed conversion ratio (FCR) tended to improve in WC (8.66 kg DMI/kg LWG) (*p* = 0.08). The highest-quality silage was produced in group WC, and it could be successfully fed to growing bulls as the sole forage.

## 1. Introduction

Due to the food–feed–fuel competition for arable land, more sustainable forage production is needed to make use of novel, alternative resources, and less fertile soils [1]. In recent years, we have witnessed a growing interest in the North American species Virginia fanpetals (*Sida hermaphrodita* L. Rusby), known in Europe as an attractive source of biomass for energy production. This perennial crop is also used as fodder for livestock, and as a source of herbaceous raw material, fiber for the textile industry, and nectar for beekeeping. Virginia fanpetals is characterized by low susceptibility to diseases and pests, high tolerance of low temperatures (up to −35 °C) and drought stress, and high phytoremediation potential. The species has low agronomic requirements, and it can be grown in soils with low OM content, producing high biomass yields of 9 to 20 t/ha dry matter (DM), depending on the time of harvest [2]. Mature Virginia fanpetals plants produce up to 30 green rigid stems, with circular cross-sections, are often hollow, 5–30 mm in diameter, and reach a height of 4.40 m at the end of the growing season; leaf blades are 300–360 mm wide and 240–280 mm long, and the length of petioles are 150–180 mm. Virginia fanpetals biomass harvested in the bud development stage has high CP content of 170–250 g/kg DM and high methane yield, which points to high OM degradation. In the bud development stage, plants reach a height of 1.5 m, with a stem diameter of 5–15 mm [2,3,4]. The proportion of leaves in plants, on a DM basis, ranges from 50% before the bud development stage to 25% at the end of the growing season. The content of CP and lignin is 308–208 g/kg DM and 56–78 g/kg DM, respectively, in leaves, and 248–44 g/kg DM and 51–139 g/kg DM, respectively, in stems, depending on the growth stage [5].

Its specific chemical composition makes Virginia fanpetals an attractive forage crop; however, Borkowska and Molas [4] demonstrated that it is not suitable for hay production or prolonged wilting due to high leaf losses. It appears that Virginia fanpetals herbage intended for ensiling could be harvested with a round baler or a forage harvester after a short wilting period, or directly with a forage harvester. During wilting, the loss of water increases the osmotic pressure; thus, inhibits the growth of Clostridium spores, increases the relative amounts of WSC [6], and limits proteolysis [7]. Slottner and Bertilsson [8] noted differences in the fermentation pattern during ensilage when the same crop was harvested with a forage harvester and a round baler. Precision chopping increased the availability of substrates for microorganisms, and fermentation was more extensive when the crop was ensiled in a steel silo.

Previous studies investigating Virginia fanpetals herbage chopped without crushing and ensiled under natural moisture conditions revealed unsatisfactory acidification, low lactic acid concentration, and high concentrations of acetic acid and butyric acid in silage; the addition of molasses and formic acid had a positive influence on silage quality [5]. A different fermentation pattern was observed by Fijałkowska et al. [9] in herbage harvested with a precision-cut forage harvester equipped with a crusher, and ensiled in high-density polyethylene (HDPE) drums. Silage without additives was characterized by extensive lactic acid fermentation and low concentrations of volatile fatty acids (VFAs). Our earlier study [10] demonstrated that Virginia fanpetals herbage intended for ensiling, harvested with a forage harvester, can be fed to beef cattle as a component of diets containing maize silage and grass silage, or as the sole forage supplemented with ground cereal grain. It was also found that Virginia fanpetals silage can be a substitute for alfalfa silage in diets for medium-yielding cows, without significantly compromising their performance [11].

Differences in the nutrient content of stems and leaves, and in the fermentation pattern of variously chopped Virginia fanpetals herbage, observed in laboratory analyses, suggest that different harvesting and preservation methods may affect the quality and feed value of silage. Thus, the aim of this study was to determine the effect of Virginia fanpetals harvesting methods on silage quality, intake and digestibility, and the performance of growing Holstein Friesian (HF) bulls fed the experimental silage as the sole forage, and to identify the optimal harvesting technique for Virginia fanpetals silage intended for feeding to cattle.

## 2. Materials and Methods

### 2.1. Treatments and Experimental Design

Virginia fanpetals silage was produced under three different technologies: direct harvesting with a precision-cut forage harvester (DC), harvesting with a precision-cut forage harvester after field wilting (WC), and harvesting with a round baler after field wilting. The experimental silage was fed for 81 days to young bulls as the sole forage supplemented with 3.0 kg of concentrate/head/day, to evaluate its intake and digestibility, the feed conversion ratio (FCR), and the growth performance of animals.

### 2.2. Silages

Virginia fanpetals biomass was harvested in the third year after planting, in a plantation established in northeastern Poland (53°05′11.2″ N, 21°11′34.1″ E) on sandy soil fertilized with N_100_ P_25_ K_65_ kg/ha. The harvest was carried out between 10:00 a.m. and 12:00 p.m. Herbage was harvested in the bud development stage, at a height of 15 cm. Two-thirds of the plantation area was mowed with a Claas Corto 270 drum mower (GmbH, Harsewinkel, Germany), and herbage was left in the field for 24 h to wilt. The remaining part of the herbage was harvested directly with a Claas Jaguar-870-Profi self-propelled forage harvester (GmbH, Harsewinkel, Germany) equipped with a 445 Kemper head and a cracker. After herbage cutting, representative samples were collected and frozen at −25 °C. After 24 h, in one part of the plantation, herbage was harvested with the INTEGRAL ROTOR round baler–wrapper combination with the 14-knife OPTICUT system (i-BIO+, Kuhn, Saverne, France), and in the other part of the plantation, herbage was harvested with the forage harvester that had previously been used for direct harvesting, after installing an adapter for harvesting crops that had been field-wilted. During each harvesting operation, NOACK AC SIL1 acidifier (Noack & Co, GmbH, Wien, Austria) was applied at 5 L/t of herbage with a Junkkari HP 5 applicator (Junkkari OY, Ylihärmä, Finland). The bales were wrapped with six layers of a 25 µm thick and 500 mm wide white plastic film (FN WRAP Plus 500), and they were stored in an upright position. The harvested plant material was piled in a heap on a concrete plate, compressed using a Manitou MLT 735 PowerShift telescopic handler-loader (Manitou BF, Ancenis, France), and covered with a 40 µm thick underlay silage film (Bag Polska Sp. z o.o., Krzemieniewo, Poland), a 120 µm thick three-layer SILO-VIT^®^M-Silo polyethylene film (RKW Hyplast, Hoogstraten, Belgium), and a silage protection mat. Chemical and morphological composition of Virginia fanpetals herbage and parameters describing its suitability for ensiling are presented in Table 1.

### 2.3. Animals and Management

The animals used in this experiment were kept in accordance with the provisions of the Act of 15 January, 2015 m on the Protection of Animals Used for Scientific or Educational Purposes [12].

The experiment was conducted at the Agricultural Experiment Station in Bałcyny (53°35′ N, 19°51′ E) in northeastern Poland, and it involved 24 Polish HF bulls at 14 ± 1.6 months of age, with an initial live weight of 440 ± 41.7 kg. Before the experiment, the animals were fed grass silage (D-value = 635 g/kg DM) and 2.5 kg of concentrate/head/day. They were housed in groups of pens fitted with the Roughage Intake Control System and the Automatic Concentrate Station (Insentec BV, Marknesse, The Netherlands). The bulls were divided into groups by the analog method, based on their body weights, and eight animals were placed in each pen, with one animal per feeding station of the Roughage Intake Control System. In each group, silage was offered once daily at 8:00 a.m., in the same amount (DM basis), which was determined based on the highest feed intake in the pre-experimental period. Silage was cut from the heap with a silage block cutter (Topstar, BVL, Germany), and forked into feeding stations by hand. Baled silage was cut and mixed using a horizontal mixer wagon (Seko, Curtarolo, Italy), and it was additionally mixed in a feed cart. Prior to silage feeding, leftovers were removed and representative samples were taken. Silage and leftover samples were collected each day at feeding, and concentrate samples were collected once a week. The concentrate was offered in the total amount of 3.0 kg/head/day divided into four equal portions, in the concentrate station. The concentrate was composed of 975:25 of ground triticale and a mineral–vitamin premix (75 g/head/day), and it contained 863 g/kg DM (DM g/kg): 935 g OM, 129 g CP, 180 g neutral-detergent fiber (NDF), 56.9 g acid-detergent fiber (ADF), 1.17 feed unit for meat production (UFM), 84 g protein truly digestible in the small intestine limited by N (PDIN); 100 g protein truly digestible in the small intestine limited by energy (PDIE). The mineral–vitamin premix for beef cattle (Cargill, Warsaw, Poland) contained (g per kg): 235 g Ca, 79 g Na, 48 g P, 28 g Mg, 500 g Fe, 2000 mg Mn, 375 mg Cu, 3750 mg Zn, 50 mg J, 12.50 mg Co, 12.50 mg Se, 250,000 IU vitamin A, 50,000 IU vitamin D3, 1000 mg vitamin E including 909 mg DL-alpha-tocopherol. Salt licks (Lisal M, LNB, Kiszkowo, Poland) were also offered to supplement minerals. Feed intake recording began after four weeks of feeding the experimental silage.

### 2.4. Measurements

The density of baled silage (kg DM/m^3^) was calculated as bale weight multiplied by DM content and divided by the volume of a cylinder with a height of 1.20 m and a diameter of 1.25 m. The density of a silage pile was calculated as the weight of a cut-out block multiplied by DM content and divided by the volume of a cuboid (1.85 m × 0.80 m × actual height in m).

Individual silage intake was calculated as the sum of daily intakes per animal, recorded in the Roughage Intake Control System. The amount of leftovers was calculated as the difference between the amount of feed offered and feed consumed, recorded in the Roughage Intake Control System. The DMI of silage was calculated based on the individual daily intake of silage and daily DM concentration in silage. The individual intake of OM, CP, and NDF was calculated based on their average daily intake and concentration in bulk weekly samples of silage and refusals. Total intake was calculated based on the daily DMI of silage and concentrate.

The animals were weighed twice on consecutive days at the beginning and at the end of the experiment, and at two-week intervals during the experiment. The FCR was calculated as daily DMI divided by average daily gain (ADG) for each animal, and expressed as kilograms of feed required for 1 kg of body weight gain.

### 2.5. Dietary Characteristics

Representative samples of silage, concentrate and leftovers were collected daily to determine DM content. Dried samples of silage and concentrate were bulked over the week and analyzed for the content of crude ash, CP, NDF, ADF, acid detergent lignin (ADL) and WSC. Fresh silage samples were also taken at weekly intervals to determine pH, the concentrations of ammonia-N, ethanol, lactic acid and VFAs, and to evaluate particle size with the use of the New Penn State Forage Particle Separator [13].

### 2.6. Digestibility

The apparent digestibility of silage was determined by the balance method on six adult Polish Merino rams, according to the Latin square model (3 × 2), as described by Sobiech et al. [14]. The apparent digestibility of diets was determined in all bulls in the last week of each feeding period using acid-insoluble ash (AIA) as an internal marker. Samples of silage, leftovers and feces were collected for five days. Feed, leftover and fecal samples were bulked over five-day periods, frozen (at −25 °C), and analyzed to determine the content of DM, ash, total nitrogen (TN) and NDF. Fecal samples were collected from each bull after each defecation event between 7:00 a.m. and 4:00 p.m. for five days. Samples collected during the day were frozen at −25 °C. As a result, a total of five representative fecal samples were obtained from each bull. The samples were composited across sampling times for each bull. After thawing, all samples were mixed and an analytical sample was collected to determine TN by the Kjeldahl method. The remaining part was dried at 60 °C for 72 h, ground to pass through a 1-mm screen, and stored for other chemical analyses. The intake of digested OM, CP and NDF was calculated based on the intake and concentrations of AIA and NDF in diets fed, refusals and feces, using the following Equation (1) [15]:

intake of digested CFC (kg/d) = intake of CFC (kg/d) × {100 − [100 × (AIAd/AIAf) × (CFCf/CFCd)]}(1)

where: AIAd = AIA concentration in the diet actually consumed, AIAf = AIA concentration in the feces, CFCf = coefficient of the concentration in the feces, and CFCd = coefficient of the concentration in the diet actually consumed.

### 2.7. Analytical Methods

Thawed samples were dried at 60 °C in Binder FED 115 (Binder, GmbH, Tuttlingen, Germany) dryers, and were ground in a mill (ZM 200, Retsch, Haan, Germany) to a 1 mm particle size. The proximate chemical composition was determined by standard methods [16] (934.01, 942.05, 954.01, 920.39, 978.10). The content of NDF assayed with heat-stable amylase and expressed exclusive of residual ash (aNDFom), ADF expressed exclusive of residual ash (ADFom) and ADL was determined by the method proposed by Van Soest et al. [17] using the ANKOM 220 fiber analyzer (ANKOM Technology Corp., Macedon, NY, USA), and WSC was determined by the anthrone method [18]. The buffering capacity (BC) of herbage was determined by the method proposed by Playne and McDonald [19].

Silage samples were also analyzed to determine pH—with a pH-meter (HI 8314, Hanna Instruments, Woonsocket, RI, USA), the content of lactic acid and ethanol by high-performance liquid chromatography (HPLC SHIMADZU), with a MetaCarb 67H P/N 5244 column (Varian, Palo Alto, CA, USA) and 0.0025 M sulfuric acid as the mobile phase, according to the detailed protocol of manufacturer. The concentrations of VFAs were determined using a Varian 450 gas chromatograph (Varian Instruments, Palo Alto, CA, USA) coupled with a flame ionization detector (FID) and a 25-m-long CP-FFAP capillary column (internal diameter-0.53 mm, the thickness of the coating film-1.0 μm), as described by Purwin et al. [11]. The content of NH_3_-N was determined by direct distillation using the 2100 Kjeltec Distillation unit (Foss Analytical A/S, Hilleröd, Denmark) after increasing the pH of the samples by adding MgO. The following CP fractions were determined: SCP (soluble crude protein), neutral detergent insoluble crude protein (NDICP) and acid detergent insoluble crude protein (ADICP), using the methods proposed by Licitra et al. [20].

### 2.8. Calculations and Statistical Analyses

DM content was corrected according to Weissbach and Strubelt [21]. The suitability of Virginia fanpetals herbage for ensiling was determined based on its chemical composition and BC, as described by Weissbach and Honig [22], by calculating the WSC/BC ratio, minimal DM (DM_min_), and fermentability coefficient (FC). The CP fractions of silage were described according to the Cornell Net Carbohydrate and Protein System (CNCPS 6.5). PA1 (ammonia) was calculated as PA1 = ammonia × (SP/100) × (CP/100), and its degradation rate (Kd) was 200%/h; PA2 (soluble true protein) was calculated as PA2 = SP × CP/100 − PA1, and its Kd was 10–40%/h; PB1 (insoluble true protein) was calculated as PB1 = CP − (PA1 − PA2 − PB2 − PC) and its Kd was 3–20%/h; PB2 (fiber-bound protein) was calculated as PB2 = NDICP − ADICP) × CP/100, and its Kd was 1–18%/h; PC (indigestible protein) was calculated as PC = ADICP × CP/100 [23,24]. Based on the chemical composition and D-value of silage, net energy (NE) content was calculated using PrevAlim 3.23 software based on the equations described by Jarrige [25]. The data on the chemical characteristics of silage, LWG, and feed efficiency were analyzed statistically by one-way ANOVA for orthogonal designs and Duncan’s test with the use of STATISTICA software (StatSoft version 13.1, TIBCO Software Inc., Palo Alto, CA, USA).

## 3. Results

### 3.1. Silage Quality

Harvesting and preservation methods affected the DM content (*p* < 0.001) and density (*p* < 0.001) of silage (Table 2). A sieve analysis of silage revealed that the particle size distribution in the Penn State Particle Separator was similar in groups DC and WC, and silage WRB had a significantly (*p* < 0.001) higher proportion of the longest particles (>19.05 mm). Silages WC and WRB had higher WSC content (*p* = 0.047), a lower total concentration of acetic acid and propionic acid (*p* < 0.001), and a lower level of NH_3_-N (*p* = 0.030). An analysis of protein fractions according to the CNCPS revealed that the proportion of fraction PA1 (*p* = 0.022) was lower in wilted silage WC and WRB than in direct-cut (DC) silage, the proportion of fraction PB1 (*p* = 0.029) was higher in silage WC than in DC and WRB, and the proportion of fraction PB2 was higher in group WRB than in DC and WC (*p* = 0.015). The harvesting method affected the proportion of fraction PC in silage (*p* < 0.001), which was highest in group WRB.

### 3.2. The Effect of Virginia Fanpetals Harvesting Method on the Performance of Beef Cattle

#### 3.2.1. Intake

The harvesting of Virginia fanpetals herbage with a forage harvester resulted in higher DMI of silage (*p* < 0.001), thus increasing DM and CP intake in groups DC and WC (*p* < 0.001), compared with group WRB. The intake of OM and NDF was higher in group WC than in groups DC and WRB (*p* < 0.001), and the average intake of OM and NDF was higher in group DC than in group WRB (*p* < 0.001) (Table 3).

#### 3.2.2. Digestibility of Diets and Animal Performance

Diets based on wilted Virginia fanpetals silages WC and WRB were characterized by higher digestibility of OM (*p* = 0.007) and CP (*p* < 0.001), and a higher D-value (*p* = 0.026), compared with silage DC. Chopping and wilting had no influence on NDF digestibility (*p* = 0.418) in group WC. However, digestible nutrient intake was highest and the FCR was lowest in this group (*p* = 0.08). The values of LWG were higher in group WC (*p* < 0.001) than in groups WRB and DC by 0.220 and 0.094 kg/day, respectively, and in group DC than in group WRB by 0.126 kg/day (Table 3).

## 4. Discussion

### 4.1. Silage Quality

The analyzed harvesting techniques were compared in terms of the effects exerted by wilting (WC vs. DC) and processing (WC vs. WRB). The absence of differences in the content of CP, NDF, and ADF in the tested silages indicates that wilting did not lead to leaf loss. Henderson [6] found that wilting increased the WSC content of silage (*p* = 0.047). The lower density of silage, observed in group DC vs. WC, resulted from DM content since the particle size distribution was similar in both silages (Table 2). Silages WC and WRB had similar DM content, but they differed significantly (*p* < 0.001) in density, which could be due to the fact that they were not chopped [26,27]. Silage density in group WRB was comparable with the values reported for ryegrass and legume-grass silages [27]. The harvesting of wilted Virginia fanpetals (WC) herbage with a forage harvester contributed to the highest acidity and the most desirable fermentation pattern. Silage DC had lower acidity (pH = 4.42), a less desirable ratio of lactic acid to acetic and propionic acid (2.1:1.0), a twice higher concentration of butyric acid (relative to silage WC), and a N-NH_3_/TN ratio pointing to secondary fermentation.

Silages harvested with a forage harvester were characterized by a lower pH, a higher proportion of lactic acid and limited production of acetic acid, and similar observations were made by other authors [28,29]. Silage WRB with considerably lower density, relative to silages DC and WC, was characterized by satisfactory fermentation, which can be attributed to its higher DM content due to wilting. This is consistent with the findings of Slottner and Bertilsson [8], who reported that baled silage with high DM content (386 g/kg) had lower concentrations of lactic acid and acetic acid than silage with high DM (333 g/kg) and low DM (230 g/kg) content made in silos.

The quality of CP in Virginia fanpetals silage, evaluated according to the CNCPS, was affected by harvesting and preservation methods. Both wilting (DC vs. WC) and chopping (WRB vs. WC) decreased the proportion of PA1 (*p* = 0.022), a fraction related to the quality of fermentation and the extent of deamination [23,24], whereas the preservation method had no effect on the proportion of fraction PA2, containing proteolysis products (*p* = 0.441) [7]. The processing of wilted herbage contributed to an increase in the most valuable protein fraction, PB1 (*p* = 0.029). The increase in PB1 in silage WC vs. WRB resulted from the fact that rapid acidification in chopped silage inhibited the activity of plant proteases [8]. The proportion of the indigestible protein fraction (PC) in silage harvested with a forage harvester was low, compared with the value noted by Guo et al. [30] in the alfalfa silage (12.3% CP). A considerable increase in the proportion of PC in silage WRB (*p* < 0.001) could be due to its lower density, which resulted in a longer aerobic phase and a rise in temperature [31].

Silages WC and WRB had higher D-values and NE concentrations (*p* = 0.002), relative to silage DC, which was associated with higher nutrient retention during ensiling [32].

### 4.2. The Effect of Virginia Fanpetals Harvesting Method on the Performance of Beef Cattle

#### 4.2.1. Intake

In the present study, DMI was significantly (*p* < 0.001) higher in silages harvested with a forage harvester, and a positive effect of chopping on the DMI of maize silage was also observed by De Boever et al. [33]. In other studies [29,34,35], the mechanical processing of corn, ryegrass, and alfalfa silages had no influence on DMI. Weigand et al. [36] found that DMI increased in response to chopping, due to a shorter chewing time, which supported considerably higher intake. The positive effect of harvesting with a forage harvester on the intake of Virginia fanpetals silage (WC and DC vs. WRB) can also be attributed to changes in the physical structure of stems and biomass homogenization, which reduced feed sorting. The above was confirmed by significantly lower DMI of silage WRB and lower NDF intake in bulls fed WRB. In group WRB, the leftovers consisted mostly of stem segments, which had an over three-fold higher content of NDF and ADL, compared with leaves (Table 1). Lower intake of DC vs. WC could result from higher concentrations of secondary fermentation products like acetic acid [37]. Differences in WC vs. WRB intake may be related to chopping, which increases feed intake by ruminants [38,39]. The values of average intake indicate that LWG was the only factor that contributed to an increase in silage intake over time [40]. The linear manner of WRB silage intake shows that feed sorting persisted also at an older age. According to Villalba et al. [41], selective feeding behavior in ruminants is linked with a dynamic interaction between palatability and the post-ingestive feedback, which is determined by the animal’s physiological status and the chemical composition of feed.

#### 4.2.2. Digestibility of Diets and Animal Performance

Lower digestibility of CP in group DC was due to the decreased synthesis of microbial protein in the rumen, resulting from a lower supply of readily fermentable carbohydrates in bulls fed silage characterized by extensive fermentation [42]. Dewhurst et al. [43] found that limited silage fermentation had a positive effect on the synthesis of microbial proteins in the rumen. The absence of differences in the proportions of the main CP fractions, i.e., PA2 and PB, which affect the efficiency of microbial protein synthesis [23,24], confirms that the energy content of silage could be responsible for differences in CP digestibility between groups WC and DC.

Broderick et al. [35] demonstrated that the mechanical processing of alfalfa, which involved maceration, improved the digestibility of OM (significant difference), CP and NDF in silage fed to lactating dairy cows and estimated net energy for lactation (NEL) increased by 5%. However, mechanical processing decreased NDF digestibility in corn silage, which was also observed in Virginia fanpetals silage (non-significant difference, *p* = 0.418). In the present study, the processing of Virginia fanpetals herbage (WC vs. WRB) had no effect on nutrient digestibility. The high digestibility and D-value of silage WRB could be due to lower silage intake, a higher proportion of concentrate, and lower NDF content, which resulted from feed sorting. In consequence, the proportion of leaves in the ration consumed by the animals increased (Table 1). In our previous study [44], the ruminal degradability of first-harvest Virginia fanpetals after 48 h of incubation was determined at 76.1%-DM, 74.6%-OM, 87.8%-CP and 54.0%-NDF. Particular attention should be paid to the high digestibility of NDF in silage harvested with a forage harvester, which resulted from a low proportion of ADL in NDF. An important role was also played by the specific physical structure of plants harvested with a forage harvester, i.e., the presence of small fragments of stem and leaf cortex and exposed, long cellulose fibers, which could stay in the rumen for a longer time, being more abundantly colonized by microbes because the fibrous portion of the plant was damaged, thus exposing more of the cell contents and leading to higher NDF digestibility [38].

The values of daily LWG determined in the current experiment were similar to those noted by Purwin et al. [45] in a study of crossbred beef bulls and steers, whereas feed efficiency was lower in the present study. In the present study, LWG values were higher in groups DC and WC than in group WRB, and comparable with those reported by Huuskonen et al. [46] in dairy bulls (aged 363–447 days) fed grass silage supplemented with a similar amount of cereal grain. The FCR in the current study was considerably higher than the average values noted by the other authors [46,47].

The differences in daily LWG are consistent with the differences in digestible nutrient intake. The intake of digestible OM and CP was 380 g (4.7%) and 50 g (3.6%), respectively, lower in group DC than in group WC, and 1510 g (18.6%) and 220 g (15.8%), respectively, lower in group WRB than in group WC. A comparison of intake with the daily nutrient requirements for young dairy bulls, according to the INRA [48] guidelines, revealed that in group WC, the intake of NE and PDI exceeded the requirements for daily LWG of 1000 g by 10% and 8%, respectively, whereas in group DC, NE intake met the requirement and PDI intake was 7% below the requirement. In group WRB, the intake of NE and PDI was 13% and 10%, respectively, lower than the animals’ daily needs, which resulted from lower DMI of silage. In group DC, the limiting factor for PDI intake was an insufficient amount of rumen fermentable OM in silage, which reduced the supply of microbial protein digested in the intestine [41]. Total DMI was linearly correlated with the LW of bulls. In general, feed intake tends to stabilize when the animals achieve higher LW, because feed intake per kg LW decreases at that time [46].

## 5. Conclusions

Silage made from Virginia fanpetals herbage harvested in the bud development stage at DM content exceeding 20% had high nutritional value and fermentation quality. Different harvesting methods affected the fermentation pattern, the physical structure of silage and, consequently, silage intake and utilization in young cattle. Wilted Virginia fanpetals herbage harvested with a round baler was characterized by a desirable fermentation pattern during ensiling, but the resulting silage was sorted and consumed selectively by the animals, which significantly decreased intake, compromised performance, and increased the FCR. Both silage quality and animal performance were optimized when wilted herbage was harvested with a forest harvester. Direct harvesting with a forage harvester with a cracker shortens the harvesting time and increases efficiency despite extensive fermentation. The results of this study indicate that structural damage to Virginia fanpetals stems during harvest is a key element of silage production technology, ensuring a desirable fermentation pattern and high silage intake by animals.

## Figures and Tables

**Table 1:** Chemical and morphological composition (g/kg DM) of Virginia fanpetals herbage and parameters describing its suitability for ensiling.

Specification	Herbage	Leaf	Steam
Proportion in plants		0.38	0.62
DM (g/kg)	188	195	182
in DM g/kg			
Crude ash	79.9	99.7	67.6
Crude protein	174	284	107
NDF	464	191	615
ADF	341	162	481
ADL	49.7	29.0	65.0
WSC	93.3		
Buffering capacity	78.4		
WSC/BC	1.19		
DMmin (%)	35.0		
FC	30.0		

DM, dry matter corrected; NDF, neutral detergent fiber assayed with heat-stable amylase and expressed exclusive of residual ash; ADF, acid detergent fiber expressed exclusive of residual ash; ADL, acid detergent lignin; WSC, water soluble carbohydrates; WSC/BC, ratio of WSC to buffering capacity (BC); DM_min_ = 45 − 8 WSC/BC (minimal DM); FC = DM (%) + 8 WSC/BC (fermentability coefficient).

**Table 2:** Silage characteristics: density, chemical composition, fermentation products, nutritional value, protein fractions, according to the CNCPS and physical structure.

Specification	Silage			SEM	*p*-Value
	DC	WC	WRB		
Dry matter (g/kg)	185 ^B^	305 ^A^	311 ^A^	19.5	<0.001
Density (kg DM/m^3^)	179 ^B^	271 ^A^	168 ^B^	5.12	<0.001
pH	4.42 ^a^	4.12 ^b^	4.62 ^a^	0.05	0.016
Composition of dry matter (g/kg DM)					
Organic matter	902	917	897	4.38	0.148
Crude protein	173	172	167	1.92	0.133
NDF	503	488	517	7.49	0.131
ADF	355	333	372	8.24	0.106
ADL	46.6	45.9	47.7	1.56	0.102
WSC	6.31 ^b^	20.4 ^a^	15.4 ^a^	2.37	0.047
Lactic acid	62.8	65.6	47.5	6.19	0.632
Acetic acid + propionic acid	30.0 ^A^	18.0 ^B^	11.9 ^C^	3.26	<0.001
Butyric acid	1.97	0.93	0.59	0.14	0.281
Ethanol	2.63	1.69	3.75	0.50	0.255
D-value	609 ^B^	653 ^A^	633 ^B^	4.74	0.002
Net energy (UFV)	0.76 ^B^	0.82 ^A^	0.81 ^A^	0.06	0.002
Protein value CNCPS (% CP)					
PA1	2.88 a	2.43 b	2.80 a	0.082	0.022
PA2	53.29	51.97	53.61	0.643	0.441
PB1	36.05 ^a^	37.70 ^a^	32.76 ^b^	0.916	0.029
PB2	3.73 ^b^	4.05 ^a^	4.60 ^a^	0.150	0.015
PC	4.05 ^B^	3.85 ^D^	6.23 ^AC^	0.337	<0.001
Particle length (g DM/kg DM)					
>19.05 mm	148 ^B^	193 ^B^	502 ^A^	42.7	<0.001
7.87–19.05 mm	418 ^A^	405 ^A^	308 ^B^	1.47	<0.001
1.78–7.87 mm	401 ^A^	365 ^A^	173 ^B^	2.76	<0.001
<1.78 mm	33.0	37.0	17.0	0.46	0.199

DC, direct harvesting with a forage harvester; WC, harvesting of wilted herbage with a forage harvester; WRB, harvesting of wilted herbage with a round baler; D-value, digestible OM in DM; SEM, standard error of the mean; significance levels: a,b—*p* < 0.05; A,B,C,D—*p* < 0.01.

**Table 3:** The effect of Virginia fanpetals harvesting method on the performance of young HF bulls.

Specification	Silage			SEM	*p*-Value
	DC	WC	WRB		
Silage intake (kg DM)	5.76 ^a^	6.12 ^a^	4.60 ^b^	0.14	<0.0001
Refusals (kg DM)	0.72 ^b^	0.39 ^b^	1.92 ^a^	0.14	<0.001
Intake (kg)					
Total dry matter	8.38 ^a^	8.74 ^a^	7.21 ^b^	0.14	<0.001
Organic matter	7.74 ^b^	8.12 ^a^	6.61 ^c^	0.14	<0.001
Crude protein	1.34 ^a^	1.39 ^a^	1.17 ^b^	0.02	<0.001
aNDFom	3.32 ^b^	3.58 ^a^	2.79 ^c^	0.07	<0.001
Net Energy (UFV)	7.40	8.05	6.75	0.11	<0.001
PDI (g)	599 ^b^	699 ^a^	585 ^b^	12.3	<0.001
Digestibility coefficient (%)					
Organic matter	73.7 ^B^	78.9 ^A^	79.9 ^A^	0.94	0.007
Crude protein	69.8 ^B^	77.1 ^A^	78.5 ^A^	1.09	<0.001
NDF	66.3	67.0	70.1	1.20	0.418
D-value (g/kg DM)	680 ^b^	734 ^a^	732 ^a^	9.48	0.026
Initial body weight (kg)	443	437	440	2.11	0.619
Live weight gain (kg/day)	0.939 ^B^	1.033 ^A^	0.813 ^C^	0.02	<0.001
Feed efficiency (kg DMI/kg LWG)	9.11	8.66	9.05	0.14	0.08

DC, direct harvesting with a forage harvester; WC, harvesting of wilted herbage with a forage harvester; WRB, harvesting of wilted herbage with a round baler; NDF, neutral detergent fiber; PDI, protein truly digestible in the small intestine; D-value, digestible OM in DM; SEM, standard error of the mean; significance levels: a,b,c—*p* < 0.05; A,B,C—*p* < 0.01.

## Data Availability

The data presented in this study are available from the corresponding author.
